# A prospective follow-up study on how long newborns are fasting in Debre Markos Comprehensive Specialized Hospital, Ethiopia, 2020

**DOI:** 10.1371/journal.pone.0268558

**Published:** 2022-08-16

**Authors:** Bekalu Kassie, Tejitu Wube, Dube Jara, Muluken Teshome, Aster Shiferaw, Sefinew Getaneh, Melaku Desta

**Affiliations:** 1 Department of Midwifery, College of Health Sciences, Debre Markos University, Debre Markos, Ethiopia; 2 Department of Public Health, College of Health Sciences, Debre Markos University, Debre Markos, Ethiopia; 3 Department of Public Health Specialist in Epidemiology, Debre Markos Comprehensive Specialized Hospital, Debre Markos, Ethiopia; University of Cambridge, UNITED KINGDOM

## Abstract

**Background:**

At birth, continuous flow of nutrients to the fetus in utero interrupted due to cut of the route /umbilical cord/. Instead of the cord, breast-mouth connection will be the next route in the extra uterine life. Nevertheless, limited data in our locality show the duration for how long immediate newborns are fasting.

**Objective:**

This study aimed to assess time to initiation of breastfeeding and its predictors among postnatal mothers within 12 hours of birth in Debre Markos Comprehensive Specialized Hospital, North West Ethiopia, 2020.

**Methods:**

A Facility based prospective follow-up study was conducted among 475 participants who were selected using systematic random sampling techniques. To collect the data, techniques including interview, chart review and observation were used. Data was entered to Epi-data version 3.1 and analyzed by STATA 14 software. A cox proportional hazards regression model was fitted to identify predictors for survival time. Results of the final model were expressed in terms of adjusted hazard ratio (AHR) with 95% confidence interval, statistical significance was declared with P-value is less than 0.05.

**Results:**

Newborns were fasting breast-milk for the median time of 2 hours. In this study, 25% of participants initiated breastfeeding within 1 hour, pre-lacteal while 75% initiated within 3 hours. Gave birth to multiple babies (AHR 0.37, 95% CI (0.19, 0.69)), operative delivery (AHR 0.77, 95% CI (0.62, 0.96)), got advice on timely initiation of breastfeeding immediately after delivery (AHR 0.79, 95% CI (0.63, 0.97)), pre-lacteal feeding initiation (AHR 10.41, 95% CI (2.82, 38.47)) and neonatal sickness (AHR 0.08, 95% CI (0.03–0.19)) were statistically significant predictors for time to initiation of breastfeeding.

**Conclusion:**

Fifty percent of mothers initiated breastfeeding within 2 hours. Most of them didn’t initiate breastfeeding based on world health organization’s recommendation, within one hour after delivery. Multiple birth, operative delivery, getting advice on timely initiation of breastfeeding immediately after delivery, giving pre-lacteal feeding and neonatal sickness were found to be predictors of time to initiation of breastfeeding.

## Introduction

One of the critical points under the safe delivery procedure in the immediate postpartum period is initiation of breastfeeding (breast-mouth connection), and it is widely accepted as helpful practice [1]. Timely breast-mouth connection prevents immediate infant mortality [2]. Optimum breastfeeding includes timely breast-mouth connection (within one hour of life), exclusive breastfeeding for six months, frequent feeding, continuous breastfeeding for 2 years and increase frequency of feeding during illness [2,3].

Through early breast-mouth connection, worldwide, more than one million newborn infants could be saved each year [4]. In developing countries alone, timely breast-mouth connection could save as many as 1.45 million lives each year by preventing respiratory and gastrointestinal diseases/infections in children [5,6]. Lancet neonatal survival series identifies breastfeeding as one effective intervention that can reduce 55 to 87% of all-cause neonatal mortality and morbidity [7]. Mothers who are not able to initiate breastfeeding during the first hour after delivery should still be supported to breastfeed as soon as possible to enable them because there is a relationship between time of initiation and health of the newborn [8–10]. There is a strong biological basis for how timely breast-mouth connection reduces the risk of neonatal mortality, both directly and indirectly [11,12]. Early breastfeeding initiation provides the infant with colostrum, which provides immunity factors such as anti-microbial and anti-inflammatory agents and providing a vital shield of protection against disease and death [12,13]. In addition, early breastfeeding during the neonatal period protects infants from exposure to pathogens that are often introduced through pre-lacteal feeds [14].

Breast milk intake also promotes the maturation of the intestines, and plays an important role in the development of the infant’s micro biome [14,15]. Early breastfeeding may reduce the risk of hypothermia and early mortality by facilitating skin-to-skin contact [16]. Beyond survival, breastfeeding could boost children’s brain development and provides protection against overweight and obesity [11]. Mothers also obtain important health benefits from breastfeeding, including a lower risk of breast cancer, ovarian cancer and type 2 diabetes [11]. The timely breast-mouth connection also facilitates continued breastfeeding, which is critical to reduce mortality risk throughout the neonatal period and beyond [14,17,18].

Despite significant evidences demonstrating many benefits of early breastfeeding initiation, there has been little global improvement in its adoption [3]. A review of data from the UNICEF 2017 State of the World’s Children report indicates that 45% of infants are breastfed within an hour of birth, with only marginal global improvement since 2003–2008 [19,20]. In sub-Saharan Africa, timely breast-mouth connection improved slightly from 46 percent in 2003–2008 to 51 percent in 2011–2016 [3]. Initiating breastfeeding after the first hour of life doubled the risk of neonatal mortality [21,22].

Different initiatives like International Code of Marketing of Breast-milk Substitutes (aka the Code), Innocent Declaration, Baby Friendly Hospital Initiative (BFHI), Millennium Development Goals, Global Nutrition Targets 2025 and Sustainable Development Goals [18,23–27] have been launched to improve breastfeeding practice. The Ethiopian government has approved and implemented the above-mentioned policies and programs to reduce infant and child mortality and morbidity related to improper breastfeeding in the country [28]. However, the rate of timely initiation of breastfeeding has failed to achieve the national Health Sector Transformation Plan [29,30] and WHO global target [31,32]. This calls for the need to study the effect of various factors affecting time to initiation of breastfeeding. However, there is limited data in the locality on time to initiation of breastfeeding and predictor assessment.

Therefore, this study aimed to assess time to initiation of breastfeeding and its predictors among postnatal mothers within 12 hours of giving birth in Debre Markos Comprehensive Specialized Hospital, North West Ethiopia, 2020.

## Methods

### Study design, area and period

A Facility based prospective follow-up study was conducted in Debre Markos Comprehensive Specialized Hospital from March 01 to April 30, 2020. The hospital is found in Debre Markos town and the town is 300 km from Addis Ababa and 265 km from Bahir Dar. The hospital was established in 1965 by Emperor H/Selassie and currently serves a population of around 5 million people in its catchment area. There are around 102 health centers, 8 primary and 1 general hospitals available in the catchment area of the comprehensive specialized hospital to which, other health institutions can refer cases for further management. In Debre Markos Comprehensive Specialized Hospital, there were 50 midwives, 1 clinical midwife specialist, 14 general practitioners, 1 emergency surgeon, and 7 gynecologists. Annually, there are about 6010 deliveries in average in the Comprehensive Specialized Hospital.

#### Population

All women who gave live birth in Debre Markos Comprehensive Specialized Hospital during the study period were taken as study populations. Mothers admitted to the intensive care unit immediately after they gave birth were excluded from the study.

#### Sample size determination

Sample size was determined by using single population proportion formula, but it was lower than the sample size calculated for predictors, which were statistically significant in different studies (Table 1).

**Table 1:** Sample size determination for time to initiation of breast-feeding among postnatal mothers within 12 hours of giving birth in Debre Markos Comprehensive Specialized Hospital, East Gojjam Zone, North West Ethiopia 2020.

No	Predictors	AOR	Estimated number of event	Total sample size	References
1	Postnatal counseling	3.7	196	293	(35)
2	Maternal residence	2.1	58	239	(36)
3	Birth order	1.44	237	432	(25)
4	Mode of delivery	5.7	11	19	(37)

From the findings on the table, birth order, as a predictor of time to initiation of breastfeeding, taken as a factor for giving the highest sample size. Therefore, the total sample size for this study with 10% nonresponse rate was 475.

#### Sampling technique and procedure

Based on the two months data just prior to the survey, 1110 mothers gave birth in Debre Markos Comprehensive Specialized Hospital. By considering this, the K value was calculated as two. Eligible study participants were selected immediately after they gave birth by systematic random sampling technique. This selection continued until getting adequate sample size. Data collectors followed each participant for 12 hours by using a structured observation checklist in every hour. Data collectors used delivery registration book as a sampling frame. Those mothers who gave birth at night were recruited early in the morning and followed both retrospectively and prospectively until obtaining the event of interest or until the end of follow-up time (12 hours). For other information, interview and chart review made along with the observation.

### Data collection tool and procedure

#### Data collection tool

A structured interviewer administered questionnaire, an observation checklist and chart review checklist compiled together as a single tool to collect data from study participants. The tool was constructed by modifying previous researches done on similar topics in Ethiopia [33], WHO [34] and other related published studies [35–37]. The first part contained socio-demographic and economic characteristics of the respondent. Second, third, fourth and fifth parts contained obstetric and health service related factors; traditional and cultural beliefs; social communication exposure and health-related factor questions respectively.

#### Data collection procedure

Data were collected through face-to-face interview, observation and chart review. Eight non-employed graduate midwives recruited as data collectors, and they were paid as per the legislation of the university. Observational data collection method was used to determine the outcome variable (time to initiation of breastfeeding) and interview and chart review were used to collect other information. The observation was participatory observation type and sampled mothers were followed from delivery to 12 hours. After getting the event of interest, data collectors counsel each mother on exclusive breastfeeding, good attachments and good positioning. Nevertheless, before the event of interest happened, not to affect the study, data collectors do nothing until 12^th^ hour of birth. However, without getting the counseling anyone left the hospital including those who did not initiate up to 12^th^ hour of life (right censored).

#### Data quality control

First, the questionnaire was prepared in English then translated to Amharic (local language) and back to English. The Amharic version of the questionnaire was used for the actual data collection. Pre-test was done on 5% of the sample before the actual data collection in Finote Selam Hospital, which is located in Finote Selam Town (the capital of West Gojjam Zone), about 80 kilometers from Debre Markos Town. Training was given about sampling technique, data handling, ethical conduct, and quality of data collection for two days for data collectors and supervisors. Each data collector checked questionnaires for completeness immediately after data collection. Supervisors and the principal investigator reviewed each tool to check for its completeness and early corrections and cleaning of the data.

### Study variables

#### Dependent variable

Time to initiation of breastfeeding.

#### Independent variables

*Socio-demographic and economic characteristics*. Maternal age, father’s age, marital status, place of residence, educational status of mother and father, occupational status of mother and father, fear of handling the newborn baby, religion, household monthly income, household head, and infant sex.

*Obstetric factors and health service related factors*. Type of pregnancy (wanted/unwanted), birth order, birth weight, type of birth, parity, mode of delivery, number of antenatal visits, counseling on TIBF by healthcare provider during ANC, counseling on TIBF at PNC and skin to skin contact of newborn to mother.

*Traditional and cultural belief related factors*. Avoidance of colostrum, pre-lacteal feeding.

*Social communication exposure*. Participation on pregnant women conference or forum and any support from family members after delivery

*Health related factors*. Maternal HIV status, mothers experiencing hypertension and neonatal sickness.

### Definitions of terms

**Pre-lacteal feeding:** is the practice of giving something other than breast milk to a neonate during the period before the mother’s milk given [38].

**Operational definitions**:

** Event**: Initiation of breastfeeding

**Censored:** If a mother does not initiate breastfeeding within the follow-up period (12 hours).

Start time: time of recruitment to the study

End time: 12 hours from birth

**Data Processing and analysis:** The data was checked FOR completeness and consistency, and then it was coded and entered into Epi-data version 3.1 and exported to STATA (version 14) for analysis. Kaplan-Meier survival together with log rank test was done to assess time to initiation of breast-feeding at specific times and to compare different independent variables. Graphical methods were used to check the Cox Proportional Hazard (PH) assumption.

Bi-variable Cox proportional hazards regression was done for each independent variable and outcome of interest to identify potentially significant variables for consideration in the multivariable cox proportional hazards regression model. Those variables with P-value ≤0.25 were selected for multi-variable Cox regression analysis. The result of the final model was expressed in terms of hazard ratio (HR) with 95% confidence intervals (CI). Statistical significance had been declared with P-value was less than 0.05.

#### Ethical consideration

Ethical clearance and support letter were obtained from Debre Markos University, College of Health Sciences ethical review committee and the support letter was submitted to Debre Markos comprehensive specialized hospital medical director Office. After detailed explanation of the purpose, risk and benefits of participating in the study, participants gave informed verbal consent before being recruited to the study. Each data collector was responsible to mark the yes/no option on the consent form, and supervisors as well as ethical committees reviewed it for any breach of ethical procedure. Participants were not open for the outcome variable measurement not to affect the research outcome. Confidentiality was maintained throughout the study. Generally, Participants’ involvement in the study was on a voluntary basis.

## Results

The total participants included in the analysis were 444, which made the response rate of 93.47%. Descriptive statistics and inferential statistics were computed.

### Socio-demographic and economic characteristics

Of the total participants, about 97.52% (n = 433) were Orthodox Christianity followers in religion. More than 71% were urban residents. Two hundred forty-two (54.50%) of mothers were primiparous and 248(55.86%) gave birth to a male (Table 2).

**Table 2:** Socio-demographic and economic characteristics among postnatal mothers within 12 hours of postpartum in Debre Markos Comprehensive Specialized Hospital, North West, Ethiopia, 2020.

Variable	Category	Frequency(N = 444)	Percent (%)
**Maternal age (year)**	15–19	14	3.15
	20–24	135	30.41
	25–29	170	38.29
	30–34	77	17.34
	≥35	48	10.81
**Mother’s religion**	Orthodox	433	97.52
	Muslim	7	1.58
	Catholic	4	0.90
**Household head**	Mother	71	15.99
	Father of the neonate	373	84.01
**Marital status**	Married	440	99.10
	Others*	4	0.90
**Maternal residence**	Rural	127	28.60
	Urban	317	71.40
**Maternal ethnicity**	Amhara	440	99.10
	Others**	4	0.90
**Mother education status**	Unable to read & write	93	20.95
	Able to read & write	31	6.98
	Primary (grade 1–8)	82	18.47
	Secondary (grade 9–12)	108	24.32
	College and above	130	29.28
**Family monthly income (ETB)**	≤1100	61	13.74
	1101–7071	262	59.01
	≥7072	121	27.25
**Maternal occupation**	Housewife	211	47.52
	Farmer	55	12.39
	Government employee	86	19.37
	Private employee	32	7.21
	Merchant	60	13.51
**Birth order**	1^st^	242	54.50
	2^nd^-3^rd^	154	34.68
	4^th^ and above	48	10.81
**Sex of child**	Male	248	55.86
	Female	196	44.14
**Fear of handling the newborn baby**	Yes	10	2.25
	No	434	97.75

*Widowed and divorced

**Oromo and Tigray.

### Obstetric and Health service related characteristics

More than 96% (428) of mothers gave birth to singleton neonates, while the remaining 16(3.60%) had delivered multiple neonates. A majority, 307(69.14%) of mothers, had delivered vaginally. More than half (57.43%) of mothers got advice on TIBF immediately after delivery (Table 3).

**Table 3:** Obstetric and health service related characteristics of postnatal mothers within 12 hours of postpartum in Debre Markos Comprehensive Specialized Hospital, North West, Ethiopia, 2020.

Variable	Category	Frequency	Percent (%)
Birth weight	Low birth weight (<2500gm)	37	8.33
	Normal birth weight (2500gm-4000gm)	407	91.67
Birth type	Singleton	428	96.40
	Multiple	16	3.60
Pregnancy type	Wanted	426	95.95
	Unwanted	18	4.05
Mode of delivery	Vaginal/normal	307	69.14
	Operation/CS	137	30.86
Time of birth	Day	271	61.04
	Night	173	38.96
ANC follow up	Yes	412	92.79
	No	32	7.21
Number of ANC	No ANC follow up	32	7.21
	1–3 ANC follow up	130	29.28
	> = 4 ANC follow up	282	63.51
Advice about TIBF during ANC	Yes	131	31.80
	No	281	68.20
Advise about TIBF immediately after birth	Yes	255	57.43
	No	189	42.57
Skin to skin contact made	Yes	299	67.34
	No	145	32.66

### Health related factors

From 444 mothers involved in this study, about 98% of women were recorded as sero-negative for HIV/AIDS and about 97% and 99% of mothers were free of hypertension and breast problems respectively. Similarly, about 95% of neonates were well immediately after birth. The fifth minute Apgar score of all newborns was in the normal range, and that is why they are included in the study.

### Median time of initiation of breastfeeding

**Fig 1:**
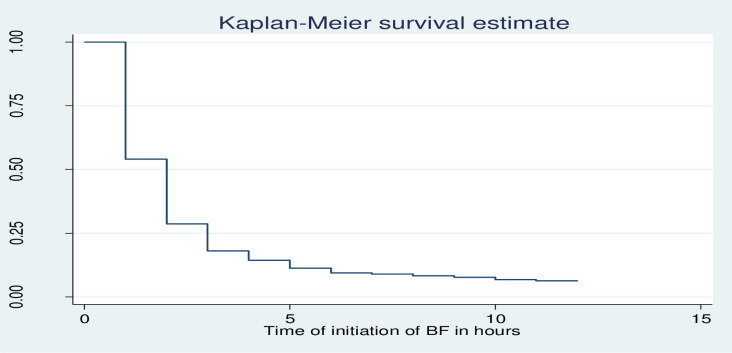
Kaplan-Meier survival estimate of delay to initiate breastfeeding among postnatal mothers within 12 hours of giving birth in Debre Markos Comprehensive Specialized Hospital, North West, Ethiopia, 2020.

Newborns in Debre Markos Comprehensive Specialized Hospital were fasting of their mothers’ breast milk for the median time of 2 hours. In addition, it was found that 25% and 75% of mothers initiated breastfeeding to their newborn within 1 hour and 3 hours of life, respectively (Fig 1). Majority of mothers ((93.92%) (95%CI 91.27%, 95.80%)) had initiated breastfeeding (experienced the event of interest) within 12 hours of follow up while the remaining, 27(6.08%), (95% CI 4.19%, 8.73%) did not initiate breastfeeding (became censored) throughout the follow-up period.

### Comparison of time to breastfeeding initiation experiences by different factors

**Fig 2:**
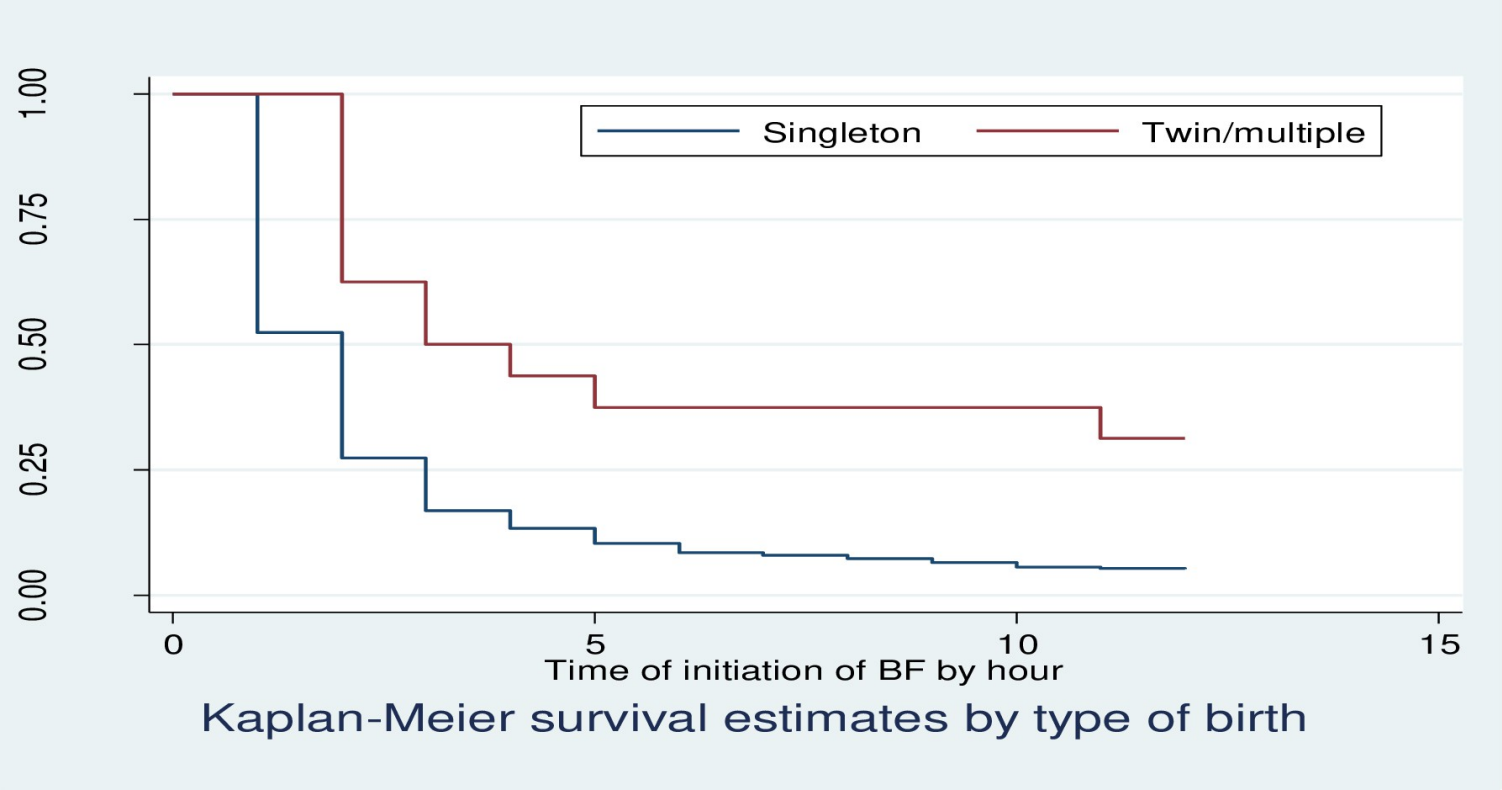
KM survival estimates by birth type among postnatal mothers within 12 hours of giving birth in Debre Markos Comprehensive Specialized Hospital, North West, Ethiopia, 2020.

Kaplan Meier survivor function curve showed that having multiple births had taken more time to initiate breastfeeding relative to singleton births (Fig 2).

**Fig 3:**
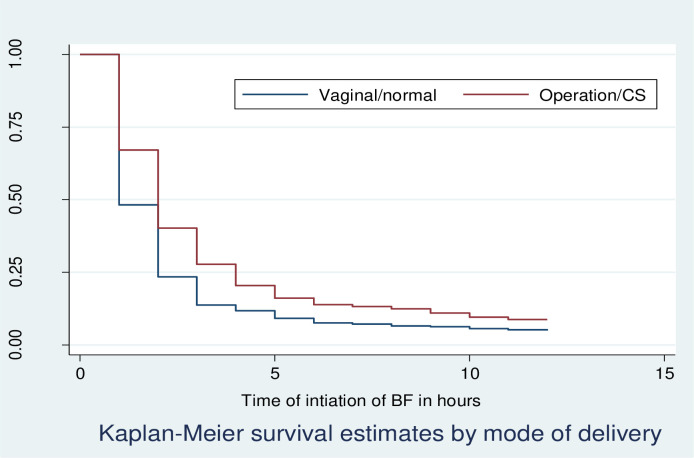
KM survival estimates by, mode of delivery among postnatal mothers within 12 hours of giving birth in Debre Markos Comprehensive Specialized Hospital, North West, Ethiopia, 2020.

Kaplan Meier survivor function curve showed that giving births operationally had taken more time to initiate breastfeeding relative to vaginally delivered mothers (Fig 3).

**Fig 4:**
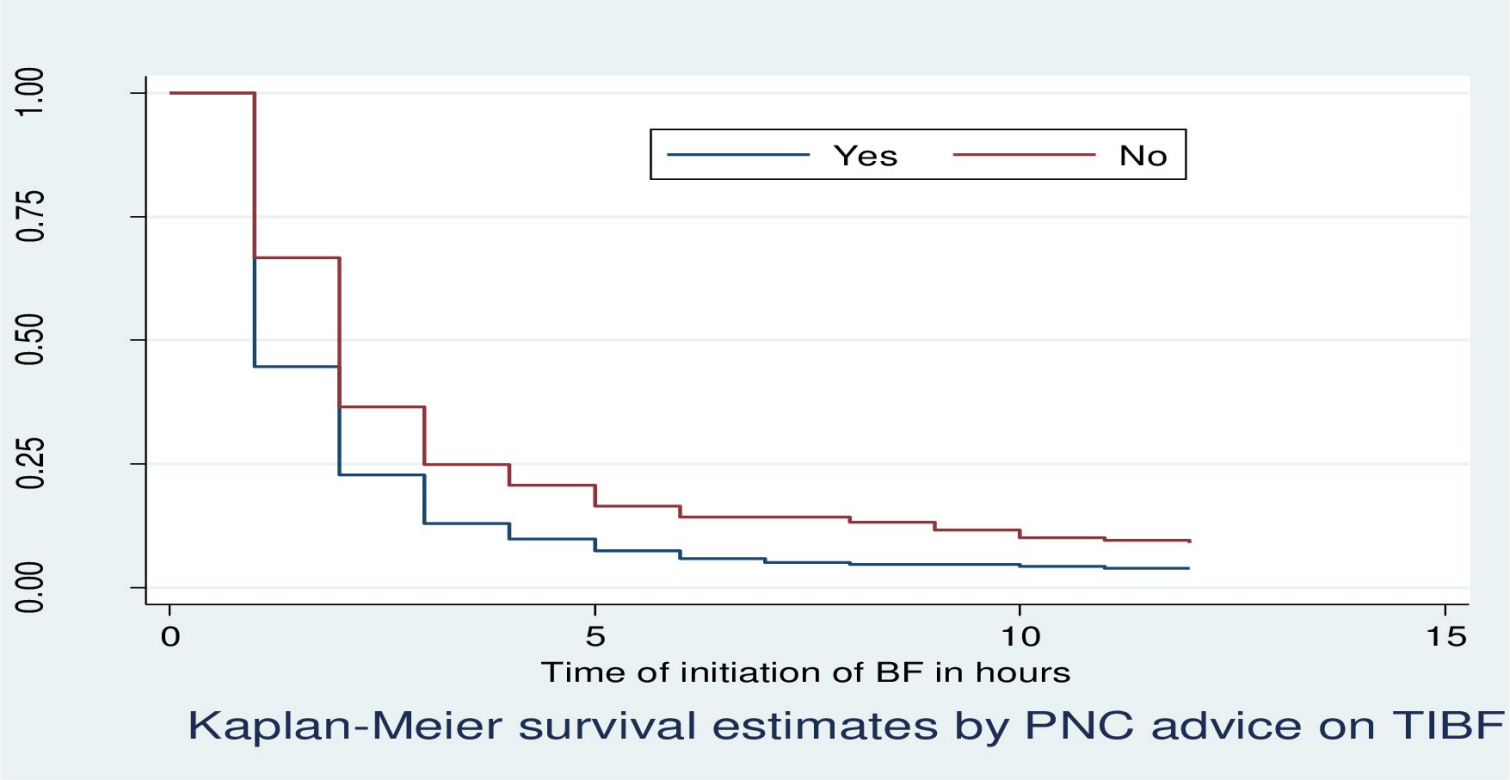
KM survival estimates by postnatal advice on TIBF immediately after delivery among postnatal mothers within 12 hours of giving birth in Debre Markos Comprehensive Specialized Hospital, North West, Ethiopia, 2020.

Kaplan Meier survivor function curve showed that mothers who did not get advice about TIBF immediately after delivery had taken more time to initiate breastfeeding relative to those who got advice about TIBF immediately after delivery (Fig 4).

**Fig 5:**
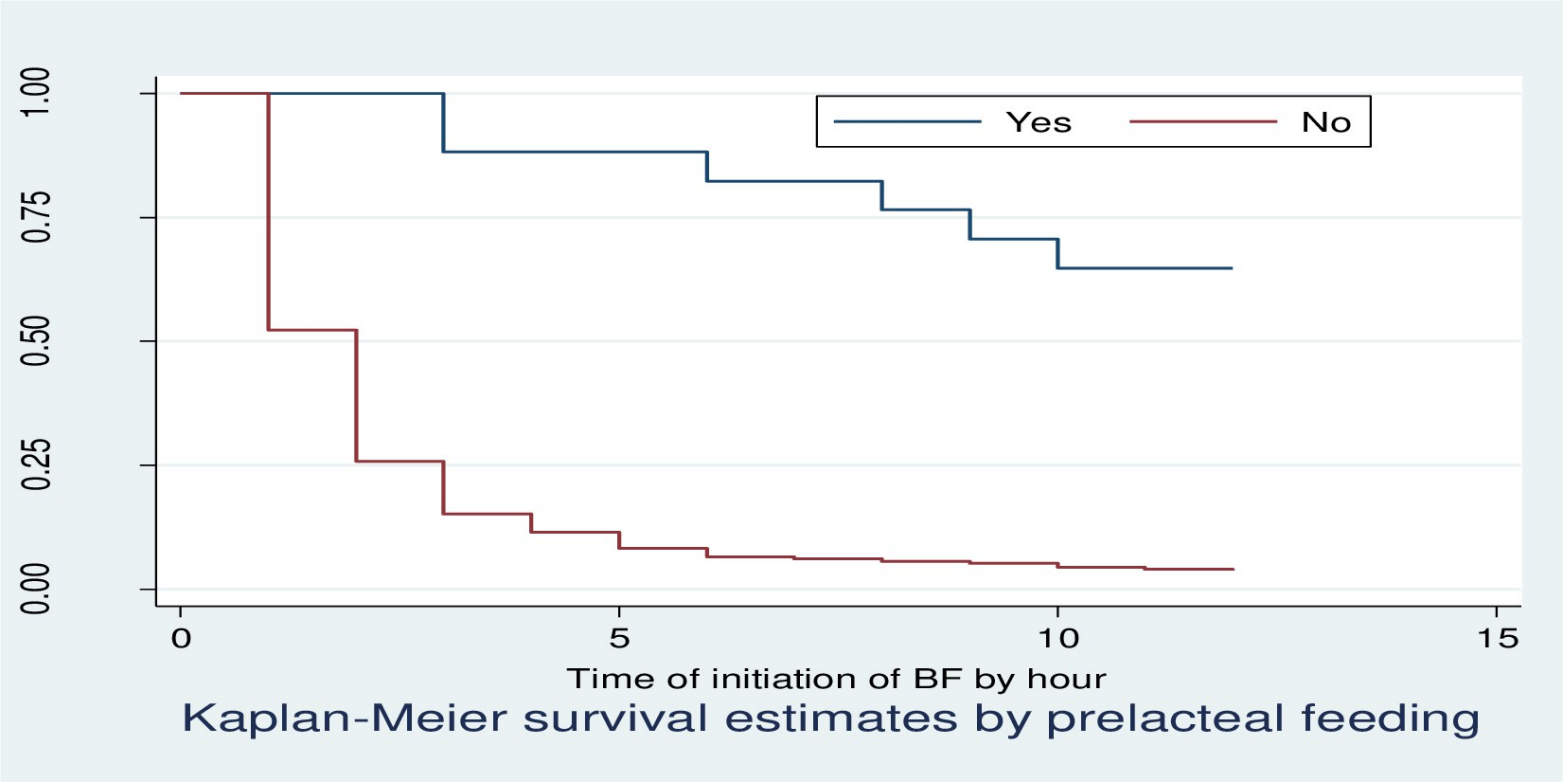
KM survival estimates by pre-lacteal feeding among postnatal mothers within 12 hours of giving birth in Debre Markos Comprehensive Specialized Hospital, East Gojjam Zone, North West, Ethiopia, 2020.

Kaplan Meier survivor function curve showed that mothers who gave pre-lacteal food had taken more time to initiate breastfeeding relative to those who didn’t give pre-lacteal food (Fig 5).

**Fig 6:**
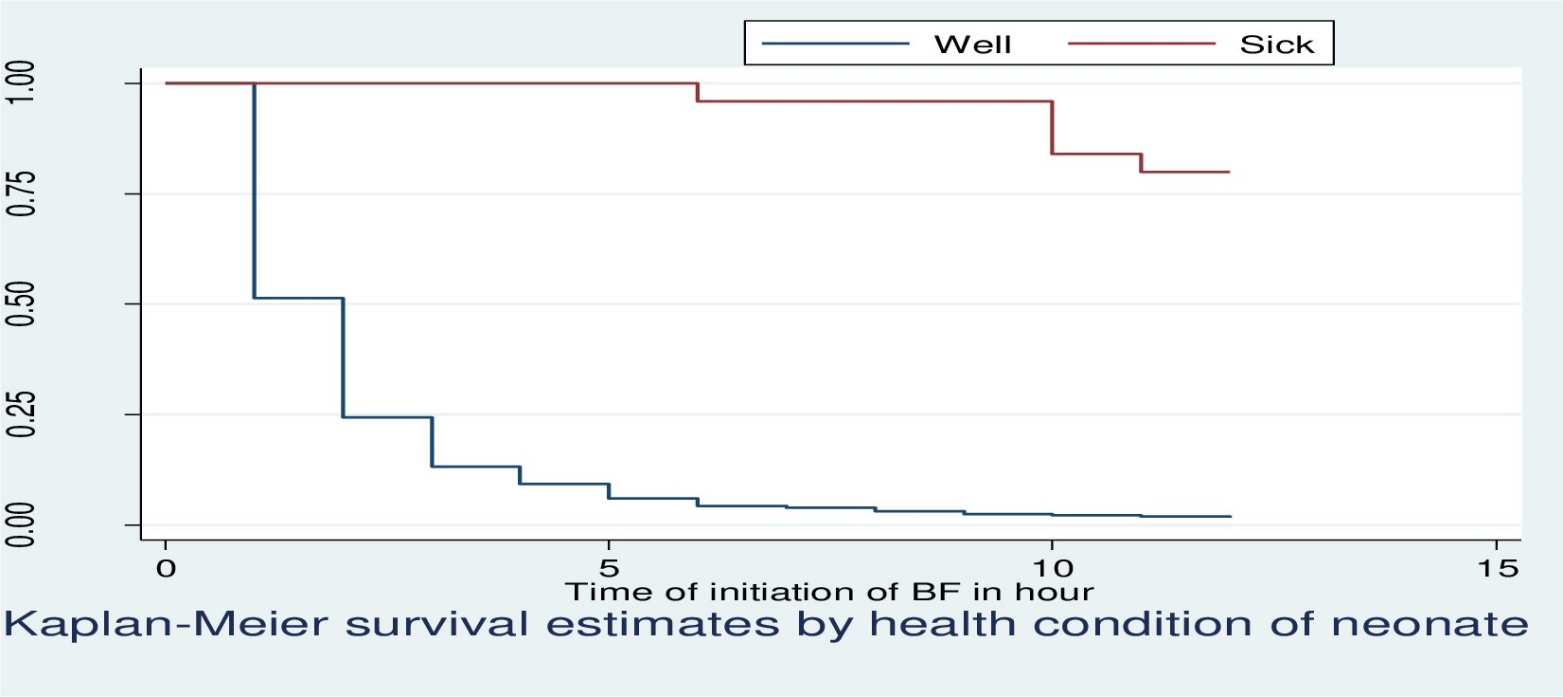
KM survival estimates by health condition of the neonate among postnatal mothers within 12 hours of giving birth in Debre Markos Comprehensive Specialized Hospital, East Gojjam Zone, North West, Ethiopia, 2020.

Kaplan Meier survivor function curve showed that mothers who had a sick neonate had taken more time to initiate breastfeeding relative to those had a well neonate (Fig 6).

The median time to initiation of breastfeeding was 2 hours for mothers who gave singleton birth and 3 hours for multiple births. Having multiple births took more time to initiate breastfeeding relative to singleton births. This result was confirmed by log-rank test showing, as there was significant difference in time to initiation of breastfeeding between singleton and multiple births.

The median time to initiation of breastfeeding was 1 hours for mothers who gave birth vaginally and 2 hours for operational delivery. The median time to initiation of breastfeeding was 1 hour for mothers who got advice about TIBF immediately after delivery and 2 hours for those who did not get advice.

For mothers who gave pre-lacteal food, the median time to initiation of breastfeeding was not observed. However, 25% of them initiated breastfeeding at 9 hours. However, the median time to initiation of breastfeeding mothers who did not give pre-lacteal food was 2 hours. Mothers who gave pre-lacteal food had taken more time to initiate breastfeeding relative to those who did not give pre-lacteal food. This result was confirmed by log-rank test showing, as there was significant difference in time to initiation of breastfeeding between mothers who gave and did not give pre-lacteal food.

The median time to initiation of breastfeeding was 2 hours for mothers who had well neonate, but the median time to initiation of breastfeeding of mothers who had sick neonate (low first minute Apgar score) was not observed. Neonate with low first minute Apgar score had taken more time to initiate breastfeeding relative to those had well score. This result was confirmed by log-rank test showing as there was significant difference in time to initiation of breastfeeding between mothers who had well and sick neonate (Table 4).

**Table 4:** Comparison of survival time to initiation of breastfeeding by log-rank test Chi-square among postnatal mothers within 12 hours of giving birth in Debre Markos Comprehensive Specialized Hospital, East Gojjam Zone, North West, Ethiopia, 2020.

Variables	Category	Median time of initiation of breastfeeding	Long rank test Chi-square	Degree of freedom	P-Value
Birth type	Singleton	2(2.35, 2.88)	13.70	1	0.0002
	Multiple	3(3.92, 8.33)			
Mode of delivery	Vaginal/normal	1(0.85, 1.15)	11.61	1	0.0007
	Operation/CS	2(1.91, 2.09)			
Advice on TIBF IAD	Yes	1	17.52	1	<0.001
	No	2			
Pre-lacteal feeding	Yes	Not observed	33.32	1	<0.001
	No	2(Not observed IQR			
First Minute Apgar score	Well	2	75.52	1	<0.001
	Low	Not observed			

#### Assessment of proportional hazard Assumption

The proportional hazard assumptions were checked based on Kaplan–Meier survival curves, which did not cross the curves in comparing two groups. Moreover, log-log plot survival probabilities were proportional across groups.

#### Predictors of time to breastfeeding initiation

Bi-variable analysis was conducted in order to see the effect of each covariate on the time to initiation of breastfeeding before proceeding to the multivariable analysis, at 25% relaxed level of significance.

In bi-variable analysis maternal residence, mother education status, family monthly average income, birth weight, birth type, mode of delivery, advise on TIBF immediately after delivery, skin to skin contact, colostrum avoidance, pre-lacteal feeding and health condition of the neonate had significant effect on time to initiation of breastfeeding among postnatal mothers at 0.25 level of significance.

Consecutively, multivariable cox-regression analysis was conducted to identify the independent effect of covariates those which shown association with outcome variable at bi-variable analysis at p-value of less than 5% significance level. Time to initiation of breastfeeding was significantly associated with birth type, mode of delivery, advice on TIBF immediately after delivery, pre-lacteal feeding and health condition of the neonate.

The rate of initiation of breastfeeding among mothers who gave multiple birth was reduced by 63% (AHR 0.37, 95% CI 0.19, 0.69) compared with those mothers who gave singleton birth. The rate of initiation of breastfeeding among mothers who delivered by operation/CS was reduced by 23% (AHR 0.77, 95% CI 0.62, 0.96) compared with those mothers who delivered vaginally. Those mothers who didn’t get advice on TIBF immediately after delivery had a 21% reduced rate of initiation of breastfeeding (AHR 0.79, 95% CI 0.63, 0.97) compared with those mothers who got advice on TIBF immediately after delivery. The rate of initiation of breastfeeding among mothers who did not give pre-lacteal food like (formula milk and cow’s milk) for their neonate was 10.41 times higher (AHR 10.41, 95% CI 2.82, 38.47) compared with those mothers who gave pre-lacteal food for their neonate. Mothers whose neonate was with low first minute Apgar score had 92% reduced rate of initiation of breastfeeding (AHR 0.08, 95% CI 0.03, 0.19) compared with mothers whose neonate was well (Table 5).

**Table 5:** Predictors of time to initiation of breastfeeding among postnatal mothers within 12 hours of giving birth in Debre Markos Comprehensive Specialized Hospital, North West, Ethiopia, 2020.

Variable	Category	Status		CHR (95% CI)	AHR (95% CI)	P-value
		Event	Censored			
Birth type	Singleton	406	22	1	1	
	Multiple	11	5	**0.42(0.23,0.76)**	**0.37(0.19,0.69)**	**0.002**
Mode of delivery	Vaginal	292	15	1	1	
	C/S	125	12	**0.75(0.61,0.93)**	**0.77(0.62,0.96)**	**0.019**
Advice on TIBF	Yes	245	10	1	1	
	No	172	17	**0.73(0.59,0.88)**	**0.79(0.63,0.97)**	**0.027**
Pre-lacteal feeding	Yes	3	9	1	1	
	No	414	18	**9.49(3.03,29.67)**	**10.41(2.82,38.47)**	**<0.001**
First minute Apgar Score	Well	412	11	1	1	
	Low	5	16	**0.08(0.03,0.19)**	**0.08(0.03,0.19)**	**<0.001**

## Discussion

The main objective of this study was to investigate how long newborns were fasting and its predictors among postnatal mothers in Debre Markos Comprehensive Specialized Hospital. The finding of the study showed that the median time of initiation of breastfeeding among mothers who gave live birth in DMCSH was 2 hours, which means 50% of mothers initiated breastfeeding within 2 hours. It was found that 25% and 75% of mothers initiated breastfeeding within 1 and 3 hours, respectively, and 93.92% had initiated breastfeeding (experienced the event of interest) within 12 hours of follow up. Birth type, mode of delivery, advice on TIBF immediately after delivery, pre-lacteal feeding and health condition of the neonate were statistically significant predictors for time to initiation of breastfeeding. Since the World Health Organization and Ministry of Health, Ethiopia are recommending to initiate breastfeeding for all neonates within one hour of life [39].

This study showed that the median breastfeeding initiation time in DMCSH was two times longer than the recommended time, which means neonates are fasting for a long time. This delay in breastfeeding may lead the neonate to hypoglycemia and other complications. Even though studies done in different parts of Ethiopia (Dembecha [40], Motta [41], Bahir Dar [42], South Gondar [33], Lalibela [39], Addis Ababa [35], Goba [36], Amibara [37], and Arsi [43]) were cross-sectional, they depict shorter breastfeeding initiation time. Similarly, studies in Nigeria, Malawi, Tanzania [38,44,45] and the Kingdom of Saudi Arabia and Bangladesh [46,47] revealed a shorter initiation time of breastfeeding. In the same way, the finding was higher than the finding of studies conducted in other countries. This variation might be due to the difference in the study participant’s recall ability of the time they initiated breastfeeding. Because the above-mentioned studies included mothers having children from 6 months to 5 years, and almost all of them were community-based studies. They did not use prospective follow-up data. On the other hand, in this study, mothers were strictly followed from delivery until 12 hours of postpartum, which can minimize recall bias.

In the current study, type of birth was statistically associated with time to initiation of breastfeeding, in which the rate of initiation of breastfeeding among mothers who gave birth to multiple newborns was reduced by 63% compared with those mothers who gave singleton birth. In multiple births, mothers and the family may perceive that feeding multiple neonates is impossible and search for other options. This searching for either formula feeding or something else may delay the initiation. This finding is in line with a study conducted in Malawi [45]. However, a study conducted in Nigeria [44] showed that birth type was not statically significant. The difference might be due to the difference in setup and sample size.

Mode of delivery was another factor statistically associated with the time to initiation of breastfeeding. It was found that the rate of initiation of breastfeeding among mothers who delivered by Cesarean Section was reduced by 23% compared with those mothers who delivered vaginally. This finding is in line with studies conducted in different parts of Ethiopia (Motta [41], South Gondar [33], Amibara [37] and Arsi zone [43]) and other countries (Uganda [48], Tanzania [38], Nigeria [44], Bangladesh [47] and Nepal [49]. The main reason for this postponement among mothers who gave birth through operation may be due to deferral of breastfeeding until maternal recovery from anesthesia, which subsequently cause a long delay in making their first contact with their infant, and mothers faced difficulty to achieve comfortable breastfeeding positions. In addition, fear of wound dehiscence is another possible justification for the delay in initiation of breastfeeding.

This study also revealed that advice on TIBF immediately after delivery was a predictor of the time to initiation of breastfeeding. Those mothers who didn’t get advice on TIBF immediately after delivery had a 21% reduced rate of initiation of breastfeeding compared with those mothers who got advice on TIBF immediately after delivery. This finding is in line with a study conducted in Tiyo woreda of Arsi zone [43], Ethiopia, Goba woreda [36], of South East Ethiopia, Addis Ababa [35] and India [50]. This might be related to the fact that mothers who did not get advice on TIBF immediately after delivery do not get the most important key messages about breastfeeding, like how to attach and position their neonate for breastfeeding and how to develop self-confidence to feed breast milk to their neonate.

In addition, giving pre-lacteal food was another predictor of the time to initiation of breastfeeding. Thus, it was found that the rate of initiation of breastfeeding among mothers who did not give pre-lacteal food to their neonates was 10.41 times higher compared with those mothers who gave pre-lacteal food to their neonates. The finding is supported by a study done at Motta town, East Gojjam zone [41]. The introduction of pre-lacteal feeds may decrease an infant’s suckling activity, which in turn can reduce the maternal milk production due to decreased breast stimulation. However, Pre-lacteal feeding was not a significant variable for timely initiation of breastfeeding in a study done at Debre Berhan, Ethiopia [39]. The possible explanation for this difference might be due to the study done at Debre Birhan was community based and included mothers interviewed who had up to six months of a child, which leads to recalling problems.

Furthermore, the health condition of the neonate was also another variable, which was significantly associated with time to initiation of breastfeeding. Mothers whose neonate was sick had a 92% reduced rate of initiation of breastfeeding compared with mothers whose neonate was without illness. There was no other study done in Ethiopia, which indicated the significance effect on time to initiation of breastfeeding. A study done in South Asia had similar finding with this study [51]. However, health condition of the neonate was not significantly associated with timely breast-mouth connection in a study done at Kingdom of Saudi Arabia [46]. The possible reasons for this difference could be related with maternal and neonatal health care differences between in this study area and Kingdom of Saudi Arabia.

### Limitation of the study

Data of mothers who gave birth at night was collected at early in the morning because data collectors face difficulty to collect the data at night. This might affect recall ability of mothers. However, it is much better than cross-sectional studies.

### Conclusion

The median time of initiation of breastfeeding was 2 hours, which is longer than the recommended time (1 hour) by WHO. Multiple birth, cesarean section delivery, mothers who didn’t get advice on timely initiation of breastfeeding immediately after delivery, giving pre-lacteal feeding and neonatal sickness were found to be predictors of time to initiation of breastfeeding. The government is better to strengthen health care facilities through endorsement of guidelines and strategies related with breastfeeding specific for multiple delivery, operational delivery, advice on TIBF immediately after delivery, pre-lacteal feeding and sick neonates. Health care providers shall give special emphasis for mothers who gave birth to multiple newborns and for those who gave birth through cesarean section.

## Abbreviations

AHR: Adjusted hazard ratio
ANC: Antenatal care
CHR: Crude Hazard ration
C/S: cesarean section
DMCSH: Debre Markos Comprehensive Specialized Hospital
E.C: Ethiopian calendar
HIV: Human Immune Deficiency Virus
PNC: Postnatal care
TIBF: Time to initiation of breast feeding

## References

[1] WHO U. Managing complications in pregnancy and childbirth: a guide for midwives and doctors– 2nd ed. Geneva: World Health Organization; 2017. Licence: CC BY-NC-SA 3.0 IGO. 2017.

[2] UNICEF. From the first hour of life. New York: United Nations Children’s Fund. 2016.

[3] Oot L, Sethuraman K, Ross J, Sommerfelt AE. The Effect of Late Breastfeeding Initiation on Neonatal Mortality: A Model in PROFILES for Country-Level Advocacy.

[4] Edmond KM, Zandoh C, Quigley MA, Amenga-Etego S, Owusu-Agyei S, Kirkwood BR. Delayed breastfeeding initiation increases risk of neonatal mortality. Pediatrics. 2006;117(3):e380–e6. 16510618 10.1542/peds.2005-1496

[5] Rudan I, O’brien KL, Nair H, Liu L, Theodoratou E, Qazi S, et al. Epidemiology and etiology of childhood pneumonia in 2010: estimates of incidence, severe morbidity, mortality, underlying risk factors and causative pathogens for 192 countries. Journal of global health. 2013;3(1). 10.7189/jogh.03.010401 PMC3700032 23826505

[6] UNICEF. UNICEF Child Info: Monitoring the Situation of Children and Woman. UNICEF: New York, NY, USA. 2012.

[7] Darmstadt GL, Bhutta ZA, Cousens S, Adam T, Walker N, De Bernis L, et al. Evidence-based, cost-effective interventions: how many newborn babies can we save? The Lancet. 2005;365(9463):977–88. 10.1016/S0140-6736(05)71088-6 15767001

[8] Organization WH. Indicators for assessing infant and young child feeding practices: conclusions of a consensus meeting held 6–8 November 2007 in Washington DC, USA: World Health Organization (WHO); 2008.

[9] Organization WH. Every newborn: an action plan to end preventable deaths. 2014.

[10] Organization WH. Guideline: protecting, promoting and supporting breastfeeding in facilities providing maternity and newborn services. World Health Organization; 2017. 29565522

[11] Victora CG, Bahl R, Barros AJ, França GV, Horton S, Krasevec J, et al. Breastfeeding in the 21st century: epidemiology, mechanisms, and lifelong effect. The Lancet. 2016;387(10017):475–90. 10.1016/S0140-6736(15)01024-7 26869575

[12] Begum K, Dewey K. Impact of early initiation of exclusive breastfeeding on newborn deaths. Alive and Thrive Technical Brief. 2010;1:99–109.

[13] UNICEF W. Capture the Moment–Early initiation of breastfeeding: The best start for every newborn. New York: UNICEF. 2018.

[14] Begum K, Dewey KG. Impact of early initiation of exclusive breastfeeding on newborn deaths. 2010.

[15] Horta BL, Loret de Mola C, Victora CG. Long‐term consequences of breastfeeding on cholesterol, obesity, systolic blood pressure and type 2 diabetes: a systematic review and meta‐analysis. Acta Paediatrica. 2015;104:30–7. doi: 10.1111/apa.13133 26192560

[16] Smith ER, Hurt L, Chowdhury R, Sinha B, Fawzi W, Edmond KM, et al. Delayed breastfeeding initiation and infant survival: A systematic review and meta-analysis. PLoS One. 2017;12(7):e0180722. 28746353 10.1371/journal.pone.0180722 PMC5528898

[17] Moore ER, Bergman N, Anderson GC, Medley N. Early skin‐to‐skin contact for mothers and their healthy newborn infants. Cochrane database of systematic Reviews. 2016(11). 10.1002/14651858.CD003519.pub4 PMC6464366 27885658

[18] Organization WH. Implementation guidance: protecting, promoting and supporting breastfeeding in facilities providing maternity and newborn services: the revised baby-friendly hospital initiative. 2018.

[19] Institute EPH, ICF. Ethiopia Mini Demographic and Health Survey 2019: Key Indicators. EPHI and ICF Rockville, Maryland, USA; 2019.

[20] UNICEF. Celebrating 20 years of the Convention on the Rights of the Child: The state of the world’s children special edition. New York: United Nations Children’s Fund Retrieved August. 2009;16:2010.

[21] Smith ER, Hurt L, Chowdhury R, Sinha B, Fawzi W, Edmond KM, et al. Delayed breastfeeding initiation and infant survival: A systematic review and meta-analysis. PLoS One. 2017;12(7). doi: 10.1371/journal.pone.0180722 28746353 PMC5528898

[22] Khan J, Vesel L, Bahl R, Martines JC. Timing of breastfeeding initiation and exclusivity of breastfeeding during the first month of life: effects on neonatal mortality and morbidity—a systematic review and meta-analysis. Maternal and child health journal. 2015;19(3):468–79. doi: 10.1007/s10995-014-1526-8 24894730

[23] Labbok M. Breastfeeding: A woman’s reproductive right. International Journal of Gynecology & Obstetrics. 2006;94(3):277–86. doi: 10.1016/j.ijgo.2006.04.008 16828483

[24] McInnes RJ, Wright C, Haq S, McGranachan M. Who’s keeping the code? Compliance with the international code for the marketing of breast-milk substitutes in Greater Glasgow. Public health nutrition. 2007;10(7):719–25. doi: 10.1017/S1368980007441453 17381952

[25] Katsinde SM, Srinivas SC. Breast feeding and the Sustainable Development agenda. Indian Journal of Pharmacy Practice. 2016;9(3):144–6.

[26] Nations U. Sustainable development knowledge platform. New Orleans: Nations U. 2016.

[27] Organization WH. Global nutrition targets 2025: breastfeeding policy brief. World Health Organization; 2014.

[28] FMOH. Ethiopian National strategy for Infant and Young Child Feeding (IYCF). 2004.

[29] Health FDRoEMo. HSTP Health Sector Transformation Plan 2015/16–2019/20 (2008‐2012 EFY). Federal Democratic Republic of Ethiopia Ministry of Health; 2015.

[30] Ethiopia FDRo. National Nutrition Programme II: 2016–2020. 2016.

[31] Zhao J, Li M, Freeman B. A Baby Formula Designed for Chinese Babies: Content Analysis of Milk Formula Advertisements on Chinese Parenting Apps. JMIR mHealth and uHealth. 2019;7(11):e14219. doi: 10.2196/14219 31782743 PMC6911233

[32] Collective GB, UNICEF, Organization WH. Global Breastfeeding Scorecard, 2017. Tracking progress for breastfeeding policies and programmes UNICEF/WHO. 2017.

[33] Mekonen L, Seifu W, Shiferaw Z. Timely initiation of breastfeeding and associated factors among mothers of infants under 12 months in South Gondar zone, Amhara regional state, Ethiopia; 2013. International breastfeeding journal. 2018;13(1):17. doi: 10.1186/s13006-018-0160-2 29743932 PMC5930958

[34] Sisay W, Edris M, Tariku A. Determinants of timely initiation of complementary feeding among mothers with children aged 6–23 months in Lalibela District, Northeast Ethiopia, 2015. BMC public health. 2016;16(1):884. 27562072 10.1186/s12889-016-3566-z PMC5000475

[35] Ekubay M, Berhe A, Yisma E. Initiation of breastfeeding within one hour of birth among mothers with infants younger than or equal to 6 months of age attending public health institutions in Addis Ababa, Ethiopia. International breastfeeding journal. 2018;13(1):4. 29410699 10.1186/s13006-018-0146-0 PMC5782370

[36] Setegn T, Gerbaba M, Belachew T. Determinants of timely initiation of breastfeeding among mothers in Goba Woreda, South East Ethiopia: A cross sectional study. BMC public health. 2011;11(1):217. 21473791 10.1186/1471-2458-11-217 PMC3088906

[37] Liben ML, Yesuf EM. Determinants of early initiation of breastfeeding in Amibara district, Northeastern Ethiopia: a community based cross-sectional study. International breastfeeding journal. 2016;11(1):7. 27064535 10.1186/s13006-016-0067-8 PMC4826535

[38] Exavery A, Kanté AM, Hingora A, Phillips JF. Determinants of early initiation of breastfeeding in rural Tanzania. International breastfeeding journal. 2015;10(1):27. doi: 10.1186/s13006-015-0052-7 26413139 PMC4582933

[39] Tilahun G, Degu G, Azale T, Tigabu A. Prevalence and associated factors of timely initiation of breastfeeding among mothers at Debre Berhan town, Ethiopia: a cross-sectional study. International breastfeeding journal. 2016;11(1):27. doi: 10.1186/s13006-016-0086-5 27729937 PMC5048691

[40] Bimerew A, Teshome M, Kassa GM. Prevalence of timely breastfeeding initiation and associated factors in Dembecha district, North West Ethiopia: a cross-sectional study. International breastfeeding journal. 2016;11(1):28. doi: 10.1186/s13006-016-0087-4 27757141 PMC5053325

[41] Tewabe T. Timely initiation of breastfeeding and associated factors among mothers in Motta town, East Gojjam zone, Amhara regional state, Ethiopia, 2015: a cross-sectional study. BMC pregnancy and childbirth. 2016;16(1):314. doi: 10.1186/s12884-016-1108-4 27756253 PMC5069976

[42] Belachew A. Timely initiation of breastfeeding and associated factors among mothers of infants age 0–6 months old in Bahir Dar City, Northwest, Ethiopia, 2017: a community based cross-sectional study. International breastfeeding journal. 2019;14(1):5. 30651748 10.1186/s13006-018-0196-3 PMC6332601

[43] Woldemichael B, Kibie Y. Timely initiation of breastfeeding and its associated factors among mothers in Tiyo Woreda, Arsi Zone, Ethiopia: A community-based cross sectional study. Clinics in Mother and Child Health. 2016;13(1):1–6.

[44] Berde AS, Yalcin SS. Determinants of early initiation of breastfeeding in Nigeria: a population-based study using the 2013 demograhic and health survey data. BMC Pregnancy and Childbirth. 2016;16(1):32. 26852324 10.1186/s12884-016-0818-y PMC4744410

[45] Nkoka O, Ntenda PA, Kanje V, Milanzi EB, Arora A. Determinants of timely initiation of breast milk and exclusive breastfeeding in Malawi: a population-based cross-sectional study. International breastfeeding journal. 2019;14(1):37. doi: 10.1186/s13006-019-0232-y 31428184 PMC6697947

[46] Ahmed AE, Salih OA. Determinants of the early initiation of breastfeeding in the Kingdom of Saudi Arabia. International breastfeeding journal. 2019;14(1):13. doi: 10.1186/s13006-019-0207-z 30984282 PMC6444675

[47] Islam MA, Mamun A, Hossain MM, Bharati P, Saw A, Lestrel PE, et al. Prevalence and factors associated with early initiation of breastfeeding among Bangladeshi mothers: A nationwide cross-sectional study. PloS one. 2019;14(4). doi: 10.1371/journal.pone.0215733 31022237 PMC6483221

[48] Kalisa R, Malande O, Nankunda J, Tumwine JK. Magnitude and factors associated with delayed initiation of breastfeeding among mothers who deliver in Mulago hospital, Uganda. African health sciences. 2015;15(4):1130–5. doi: 10.4314/ahs.v15i4.11 26958013 PMC4765405

[49] Acharya P, Khanal V. The effect of mother’s educational status on early initiation of breastfeeding: further analysis of three consecutive Nepal Demographic and Health Surveys. BMC Public Health. 2015;15(1):1069. doi: 10.1186/s12889-015-2405-y 26482789 PMC4610048

[50] Sharma A, Thakur PS, Tiwari R, Kasar PK, Sharma R, Kabirpanthi V. Factors associated with early initiation of breastfeeding among mothers of tribal area of Madhya Pradesh, India: a community based cross sectional study. Int J Community Med Public Heal. 2016;3:194–9.

[51] Sharma IK, Byrne A. Early initiation of breastfeeding: a systematic literature review of factors and barriers in South Asia. International breastfeeding journal. 2016;11(1):17. doi: 10.1186/s13006-016-0076-7 27330542 PMC4912741

